# Enucléation d’un kyste hydatique du muscle psoas chez un enfant

**DOI:** 10.11604/pamj.2019.32.3.14369

**Published:** 2019-01-03

**Authors:** Hicham Abdellaoui, Youssef Bouabdallah

**Affiliations:** 1 Service de Chirurgie Viscérale et Urologie Pédiatrique, CHU Hassan II-Fès, Faculté de Médecine et de Pharmacie, Université Sidi Mohamed Ben Abdallah, Fès, Maroc

**Keywords:** Kyste hydatique, psoas, enfant, énucléation, Hydatid cyst, psoas, child, enucleation

## Abstract

Hydatid cyst of the psoas muscle is exceptional and manifests itself as a non-specific mass syndrome. Pain or compressive symptoms lead patients to go to their doctor. This cyst is characterized by an insidious development, which explains why they can grow large. Abdominal ultrasound is the gold standard test. CT scan is better in detecting the relations between this cyst and its neighboring structures. Surgery is the therapeutic method of choice. Extraperitoneal approach helps prevent intra-abdominal contamination. Perikystectomy should be performed or, even better, enucleation. Puncture, evacuation and sterilization of the cyst are even more effective because the cyst develops in the full thickness of the psoas muscle. Too large an excision may compromise functional outcome. Prognosis is generally good. We here report the case of a 5-year old girl of rural origin presenting with a 3-month history of painful mass in the right iliac fossa characterized by a progressive and insidious development. Ultrasound showed anechogenic cystic lesion in the right iliac fossa measuring 55*38 mm, suggesting hydatid cyst (A). Scan (B) confirmed the diagnosis of hydatid cyst of the iliopsoas muscle (measuring 52*42*55 mm) associated with thinned iliac bone. Extra-peritoneal oblique mini-laparotomy was performed. Then, enucleation of the cyst without rupture was performed (C and D). Clinical and ultrasonographic follow-up was satisfactory.

## Image en médecine

Le kyste hydatique (KH) du psoas est exceptionnel et se manifeste par un syndrome de masse sans aucune spécificité. La douleur ou les signes compressifs amènent le patient à consulter. L’aspect insidieux du développement explique les tailles importantes que peut prendre le kyste. L’échographie abdominale est l’examen fondamental. La TDM permet une meilleure étude des rapports du kyste avec les structures avoisinantes. La chirurgie reste la méthode thérapeutique de référence. La voie d’abord extra-péritonéale est souhaitable afin d’éviter toute contamination intra-abdominale. L’idéale est de réaliser une périkystectomie ou mieux encore une énucléation. Aussi la ponction, l’évacuation et la stérilisation du kyste sont d’autant plus efficaces que le kyste se développe en pleine épaisseur du muscle psoas. L’exérèse trop large peut compromettre les résultats fonctionnels. Le pronostic est généralement bon. Il s’agit d’une fillette de 5 ans, d´origine rural, qui consulte pour une masse douloureuse de la fosse iliaque droite apparue de façon progressive et insidieuse depuis 3 mois. Une échographie avait mis en évidence une lésion kystique de la FID de 55 x 38 mm à contenu anechogène évoquant un kyste hydatique (A). Le scanner (B) avait confirmé le kyste hydatique du psoas iliaque de 52x42x55 mm avec amincissement de l’os iliaque en regard. La patiente a été opérée par une mini laparotomie oblique extra-péritonéale. Une énucléation sans rupture du kyste a été réalisée (C et D). Le suivi clinique et échographique était satisfaisant.

**Figure 1:**
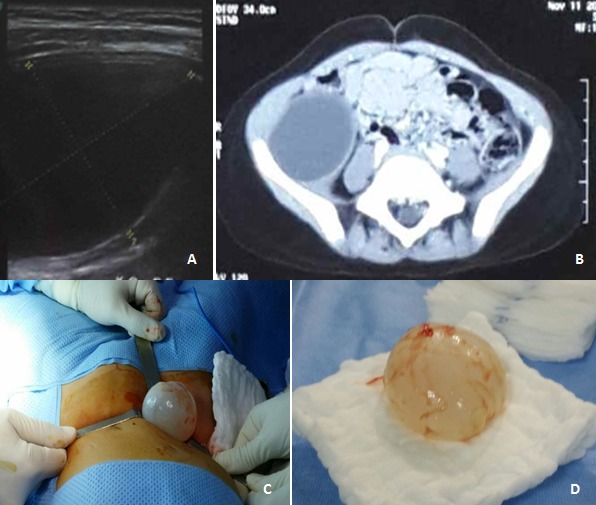
A) image echographique du kyste; B) coupe scannographique de l’image kystique du muscle psoas droit; C) image peropératoire de l’enucléation du kyste hydatique; D) aspect final sans rupture après énucléation

